# Supplementary material for: Impact of an integrated intervention package during preconception, pregnancy, and early childhood on inflammation, IGF-1, IGFBP3 during first 6 months of life: Findings from the WINGS randomized controlled trial
Source: PLOS Glob Public Health. 2025 Dec 15;5(12):e0005118. doi: 10.1371/journal.pgph.0005118 (PMC12704893; doi:10.1371/journal.pgph.0005118)
Supplement: S1 Text — (PDF) [file pgph.0005118.s001.pdf]

Impact of an integrated intervention package during preconception, pregnancy, and early childhood on biomarkers of infant growth in the first 6 months of life: A sub study in WINGS

Problem Statement and Rationale

The high burden of undernutrition is an issue of concern in Southeast Asia. Growth faltering is often seen during intrauterine life, in the 0-6 and 6-24 months periods (1-3). Understanding the physiological risk factors in each of these periods is important. My research focus is on 0-6 months age infants not only because of the recent evidence on growth faltering during this period, but also that the pathophysiology associated with growth failure after 6 months of age, may begin in early life (4). Besides, the effect of interventions that improve growth in early infancy, may persist beyond 6 months of infant age when stunting (length for age z score, LAZ <-2SD) and wasting (weight for length z score, WLZ <-2SD) increase rapidly in Southeast Asia.

The questions for my research are related to reduction in growth faltering between birth to 6 months of age. Incident stunting and wasting during this period of exclusive breastfeeding are of great concern (5). High rates of undernutrition among 0-6 months old infants were also reported in the National family Health Survey-4 (NFHS-4, 2015-16) (6). Data from an ongoing intervention study in Delhi [CTRI/2017/06/0089080] within which the current work will be embedded, show that of infants born in the study communities, 30-35% have inadequate weight gain (<15th centile WHO weight velocity) and 20-25% inadequate length gain (<15th centile WHO length velocity) between 4-6 months of age (7). This may be the beginning of stunting and wasting that continues beyond 6 months of age, when complementary foods are introduced.

The unanswered questions are why does growth falter in a third of breastfed babies between the ages of 4-6 months? Will an integrated intervention package targeted at reducing growth faltering in the first half of infancy, improve biomarkers associated with growth failure?

Infant size and gestation at birth, breast milk composition, systemic inflammation, gut microbiome structure, Insulin like growth factor -1 (IGF-1) or infant enteric and other infections could be important determinants of growth failure during early infancy (4). Studies have shown that maternal nutritional status and dietary intake influence breast milk macro and micronutrients beyond 3 months of age (8, 9). This raises the possibility that high-quality nutrition supplements for mothers may help in improving infant growth. Evidence suggests that some critical breast milk micronutrients for growth like vitamin B12, A, and D improve through maternal supplementation (10). Furthermore, in a study of Zimbabwean mother-infant pairs, systemic inflammatory markers (C-reactive protein) were persistently higher in stunted compared to non-stunted infants. This association was observed as early as at 6 weeks of age, suggesting that inflammation plays an important role in growth in early infancy (11). The gut microbiome diversity in early infancy is primarily influenced by the species involved in the metabolism of human milk oligosaccharides (HMOs) (12). Evidence suggests that HMOs interact with infant gut microbiota to regulate growth during early infancy (13). Further, HMOs containing sialic acid or fucose by preventing adhesion of pathogenic microorganisms to intestinal epithelial surfaces protect infants from enteric infections (14). Mothers of stunted infants showed a lower abundance of sialylated HMOs in breast milk at 6 months of age (15). The succession of early-life microbiome plays an important role in growth, maturation of endocrine and mucosal immune function (13).Bifidobacterium longum and Faecalibacterium prausnitzii are the most abundant taxa in healthy infants (16, 17). Insulin-like growth factor 1 (IGF-1) through short chain fatty acid (SCFA) metabolism may also play an important role in early-life growth (18). As inflammation in early life influences IGF-1 level, it can be postulated that 'turning off' the infant inflammatory axis may increase IGF-1 levels.

Our organization The Society for Applied Studies (SAS) is conducting an individual randomized controlled study (WINGS – Women and Infants Integrated Growth Study) which aims to measure the impact of an integrated package of interventions (health, nutrition, psychosocial care and support and water, sanitation, and hygiene (WASH)) delivered during preconception, pregnancy and early childhood on preterm, small for gestational age (SGA), and stunting at 24 months of age (7). In this study which uses a factorial design, non-pregnant women of reproductive age were randomized to receive the preconception package of interventions or to routine care. Once women are identified to be pregnant, they are randomized again to either receive the pregnancy and early childhood package of interventions or to routine care. The intervention package is expected to improve breast milk quality, reduce systemic inflammation, promote healthy microbiome assembly, and increase IGF-1. The pathway through which the interventions may reduce growth faltering are shown in Figure 1.

Figure 1 Postulated pathways of intervention effect to reduce growth faltering first 6 months of age

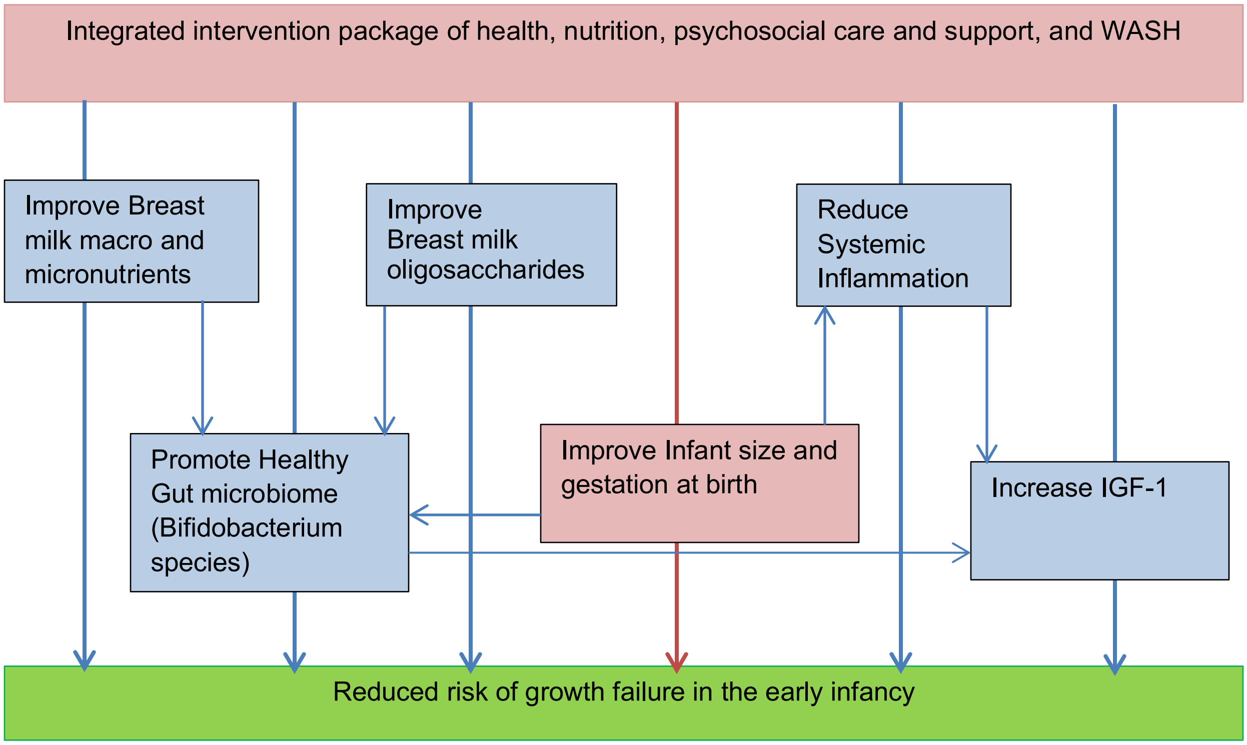

This study provides an excellent opportunity to measure biomarkers (breast milk quantity and quality, infant systemic inflammation markers, gut microbiome, and IGF- 1) of growth failure in first 6 months of life; these are currently not being measured in WINGS (4). Including these assessments t in WINGS will generate new knowledge on whether the integrated intervention package improves biomarkers related to early growth faltering and if so, what are the mechanisms through which this is achieved. This study is an attempt to link mechanistic science to the primary trial that is currently measuring only clinical outcomes.

Hypothesis

Intervention effect

An Integrated package of interventions (health, nutrition, psychosocial care and support and WASH) throughout the preconception, pregnancy and early childhood periods will improve breast milk quality (macro and micronutrients, oligosaccharides), reduce systemic inflammation markers (CRP, AGP), promote healthy gut microbiome (Bifidobacterium species) and increase IGF-1 at 6 months of age compared to routine care.

Mediation effect

The effect of the integrated package of interventions on growth failure during 3-6 months of age will be mediated through infant size and gestation at birth, breast milk quality, infant gut microbiome, systemic inflammation markers, and IGF-1 measured at 3 months of age.

Objectives

Primary

- To determine effect of an integrated package of interventions (health, nutrition, psychosocial care and support and WASH) throughout the preconception, pregnancy and early childhood periods on breast milk quality (macro and micronutrients, oligosaccharides), systemic inflammation markers (CRP, AGP), gut microbiome (Bifidobacterium species) and IGF-1 at 6 months of age compared to a group getting routine care

Secondary

To quantify the mediating effect of

- Infant size and gestation at birth
- Breast milk quality (macro and micronutrients, oligosaccharides) measured at 3 months of age
- Gut microbiome (Bifidobacterium species) measured at 3 months of age
- Systemic inflammation (CRP, AGP) measured at 3 months of age
- IGF-1 measured at 3 months of age

of the package of interventions on growth failure during 3-6 months of age.

Biological markers and their time points of assessment in the proposed study

			Infant age	
**Biological markers**	**Biological sample**	**Volume**	**3 months**	**6 months**
**Breast milk Macro** (% fat, % protein) **and micronutrients** (vitamin A, vitamin B12, vitamin D)	Breast Milk	30 ml	√ (n=400)	√ (n=400)
**Breast milk Oligosaccharides** [2′-fucosyllactose (2′FL), 3-fucosyllactose (3FL), lacto-N-tetraose (LNT), lacto-N-fucopentaose I (LNFP I) and 3′-Sialyllactose (3′SL), 6′-Sialyllactose (6′SL)]				
**Systemic inflammation markers** (CRP, AGP)	Infant blood	3 ml	√ (n=400)	√ (n=400)
**Growth-related hormones** (IGF-1, IGF-BP3)				
**Gut microbiome**	Infant Stool	5 gm	√ (n=400)	√ (n=400)

Ongoing work which has led to the project

I am an investigator in the ongoing WINGS. The study is supported by the Biotechnology Industry Research Assistance Council (BIRAC), Department of Biotechnology (DBT), Government of India and the Bill & Melinda Gates Foundation (BMGF) [CTRI/2017/06/0089080]. In this study, 13500 women of reproductive age group have been enrolled and randomized to receive the preconception intervention package or to routine care, until they are identified to be pregnant. Pregnant women are randomized either to receive the pregnancy and early childhood intervention package or to routine care. The package of interventions includes health care for growth-relevant conditions, nutrition, WASH and psychosocial care and support. The interventions were selected based on their effect on adverse pregnancy outcomes (low birth weight or preterm birth or SGA) or on stunting in the first two years of life.

I was the first Young Investigator who joined the Young Investigator Programme (http://www.prernaindia.in/) in SAS in 2014. I have now graduated from the programme and will independently develop research projects within this seminal intervention study (WINGS). The proposed study gives me a unique opportunity to grow into an independent researcher, building on my ongoing observations from the WINGS.

Methods

Study Setting

The setting for WINGS are low-to mid-socioeconomic neighborhoods in South Delhi, India (7).

Figure 2 Design of the WINGS Trial

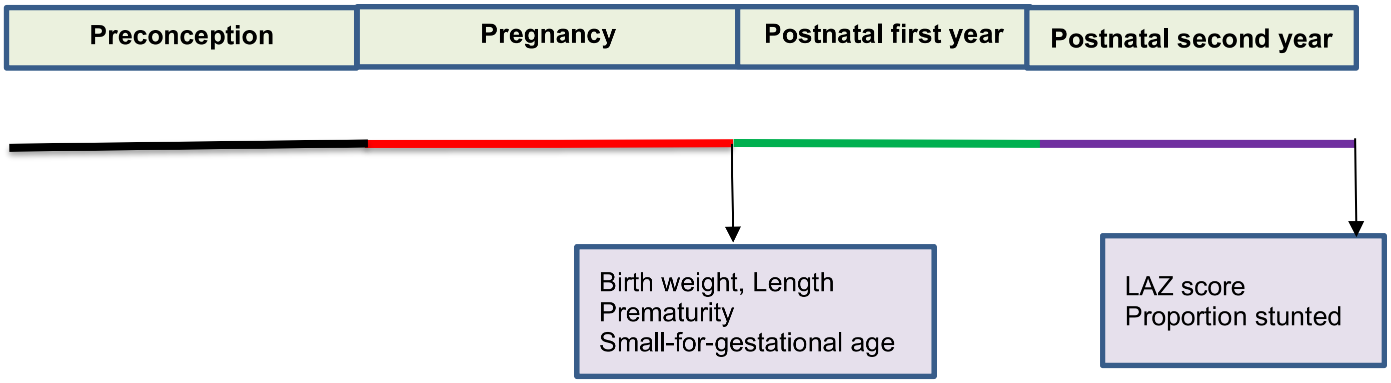

The factorial design will result in 4 Groups: Group A (Preconception + Pregnancy and Early childhood intervention); Group B (Only Preconception intervention); Group C (Only Pregnancy and Early childhood intervention); Group D (Routine care throughout)

Mother-infant pairs for the proposed study will be selected from Groups A and D.

Study interventions

The intervention package is summarized in Table 1

Table 1 Package of interventions in WINGS

<table>
<tr><th rowspan="2">Period</th><th colspan="4">Intervention group</th><th rowspan="2">Control group</th></tr>
<tr><th>Health</th><th>Nutrition</th><th>Psychosocial care</th><th>WASH</th></tr>
<tr><td>Preconception</td><td>Manage medical conditions like reproductive tract infections (RTI), Tuberculosis, Thyroid disorders, pre-diabetes, and diabetes</td><td>Manage undernutrition and anemia,
Provide iron-folic acid (IFA), multiple micronutrients, snacks, egg, or milk</td><td rowspan="3">Promote positive thinking for all, Manage depressive symptoms</td><td>Promote menstrual, personal and hand hygiene</td><td rowspan="4">Care from routinely sought sources- government (free of cost) and private providers</td></tr>
<tr><td>Pregnancy</td><td>Promote at least 8 antenatal visits, Manage medical conditions like thyroid disorders, gestational diabetes mellitus, gestational hypertension, asymptomatic bacteriuria, RTI, Provide calcium and vitamin D supplementation</td><td>Manage undernutrition and anemia, Provide IFA, multiple micronutrients, snacks and milk, Monitor weight</td><td rowspan="2">Provide water filters, soap, disinfectant and install hand washing station</td></tr>
<tr><td>Mothers (first 6 months of infant age)</td><td>Facilitate postnatal checkup on day 42 postnatally</td><td>Provide IFA, multiple micronutrients, calcium and Vitamin D, snacks, and milk</td></tr>
<tr><td>Early Childhood</td><td>Educate caregivers to identify danger signs and seek early care for illness</td><td>0-6 m: Counselling and support for early initiation and exclusive breastfeeding

6-24 m: Counselling and support timely complementary feeding and continued breastfeeding, Provide milk cereal mix, Monitor weight gain</td><td>Counselling, and demonstration for caregivers on early child play and stimulation</td><td>Provide potty and play mat</td></tr>
</table>

Sample Size

Sample size for intervention effect

There are very few intervention studies that have assessed these biomarkers in the first 6 months of life (19). In WINGS, we assumed a 0.2 SD increase for LAZ score and a relative reduction of 25% in stunting at 24 months in preconception, pregnancy, and early childhood care group (Group A) compared to control (Group D). For the proposed study, we hypothesize that the package of interventions will improve biomarkers of growth failure and have assumed a similar or higher effect size. We have assumed a 50% higher effect size i.e. 0.3 SD (1.5 times of 0.2 SD) for continuous outcomes (breast milk oligosaccharides, infant systemic inflammation markers, IGF-1) and 25% relative improvement for binary outcomes (infant gut microbiome).

All sample size calculations are based on 80% power and 95% confidence levels.

Effect of preconception, pregnancy and early childhood care compared to routine care (Groups A vs D)

	**Effect size**	**Sample size per group**
**Systemic inflammation**<br>CRP	0.30 SD (1.4 ng/mL; 1 SD=4.7 mg/L)	176
**Gut microbiome analysis**<br>(Bifidobacterium species) relative abundance (60%)	25% relative increase	152
**Growth-related hormones**<br>Mean IGF-1	0.30 SD (4.9 ng/mL; 1 SD=16.3 ng/mL)	176
**Breast milk**<br>3-fucosyllactose	0.30 SD (70 µmol/mL; 1 SD= 232 µmol/mL)	176

Assuming 15% caregivers will refuse sampling, we aim to enroll 200 infants in the two groups (Groups A and D) i.e. total 400 infants at 3 and 6 months of age.

Sample size for the mediation effect

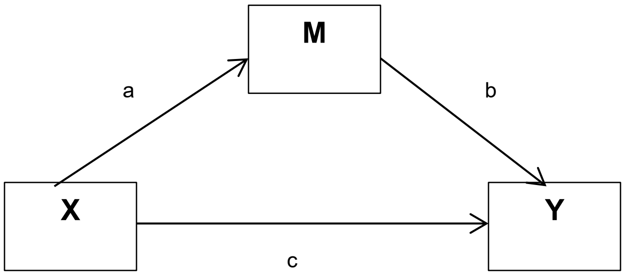

Figure 3 The Mediation model

Figure 3 shows a mediation model which is presented in regression equation 1 and 2

$$M=aX + €_1 \quad (1)$$

$$Y= cX + bM + €_2 \quad (2)$$

The direct effect of X on Y is indicated as c. The indirect effect of X on Y is through M(a), and the effect of M on Y controlling for X (b). The total effect(C) is the sum of the direct effect c and indirect effect ab.

With a sample size of 400 infants, we will have at least 80% power to detect an indirect effect of 0.06 SD using the Monte Carlo power analysis, 5000 replications, 20000 Monte Carlo Draws per replication and 95% confidence level (20).

Ethical Considerations and Informed Consent

Ethical approvals will be obtained from the ethics review committee of the Society for Applied Studies. Written informed consent will be obtained from both parents for participation in this study.

Status of the main trial (WINGS)

The enrolment in the preconception period (13500 participants) has been completed. Till 24th April 2020, a total of 4271 women were randomized (second randomization) to pregnancy and early childhood care interventions or to routine care. 3163 out of expected 4400 live births have occurred and 2073 infants are over 6 months of age.

A pertinent issue for this application is that all infants in WINGS will be older than 6 months by the time the fellowship is scheduled to start i.e. 1st September 2021. The sample collection, therefore, would need to be done earlier. I have discussed this with my supervisor, mentors, and others in the host institution. We all feel that the questions proposed are very important to understand the pathophysiology of growth failure in early infancy and the ongoing WINGS provides a unique opportunity to answer these. SAS has, therefore, decided to go ahead with the study and sample collection using internal resources. The fellowship funds (if I qualify) will be very important for covering costs for sample analyses and for my training.

Outcome assessment

For the proposed study, 200 consecutively born infants and their mothers from the preconception, pregnancy, and early childhood care group (Group A) and control group (Group D) will be selected. A statistician at the World Health Organization who generated the randomization list for the WINGS will communicate the Participant identification numbers of the mother-infant pairs to be enrolled in this study.

Digital weighing scales (model 354, to the nearest 10 g) and infantometers (model 417, to the nearest 0.1 cm) manufactured by Seca, California, USA are being used by trained workers for weight and length measurements at infant ages 3 and 6 months (21, 22). Information on breastfeeding practices and caregiver-reported morbidity are also being collected. Additional measurements (Breast milk macro and micronutrients, oligosaccharides, Infant systemic inflammation markers, gut microbiome, and IGF-1) will be done for this study. These are currently not a part of the WINGS. Details of the variables are summarized in Tables 3 and 4.

Biological Sample Collection, Transportation and Testing

Breast milk

A standard protocol for breast milk collection will be implemented to collect breast milk after complete expression of milk using a hand expression from one breast. The breast milk sample will be homogenized and 15-30 mL from a full breast expression will be chilled immediately on ice pack (-20C).The remaining milk will be returned to mother in a cup for immediate consumption by the infant Milk will be aliquoted into smaller containers and stored at -80°C the “Clinical and Research laboratory” in SAS. Breast milk micronutrients (vitamin A, B12, D) and breast milk macronutrients (% fat, % protein) will be analysed according to established protocols (23-25). Milk oligosaccharides will be analysed in a targeted quantitation by using ultra performance liquid chromatography triple quadrupole mass spectrometry (UPLC/QQQ-MS) in multiple reaction monitoring (MRM) mode (26). The breast milk analysis will be done at St. John’s Research Institute, Bangalore, India. The methods for analysing breast milk samples have been summarized in Table 2.

Table 2 Methods of analysing breast milk samples

Breast milk Composition	Methods
Fat	Gas chromatography, coupled to a flame ionization detector (GC-FID)
Protein	Spectrophotometer
Vitamin A	High-performance liquid chromatography
Vitamin B12	Liquid Chromatography with tandem mass spectrometry
Vitamin D	Liquid Chromatography with tandem mass spectrometry
Oligosaccharides	UPLC/QQQ-MS in MRM mode

Infant stools

~5 gm stool samples will be collected from infants at 3 and 6 months of age in OMNIgene GUT kit (27). The stools will be transported from the field the “Clinical and Research laboratory” in SAS at 4°C on ice packs and will be kept in -80°C until DNA extraction. Microbial DNA extraction will be performed from 200-250 mg stool using standard microbial DNA isolation kits and will be stored at − 20°C. Microbial DNA isolated from the stool will be further subjected 16s rRNA gene amplicon sequencing using universal primers (V3-V4) and sequenced data will be analysed using the QIIME (v 1.9.1) pipeline. Initial quality control and checks will be performed to remove ambiguous bases, homopolymers and short fragments from the sequenced reads. Chimeric sequences (hybrids of multiple parent sequences falsely interpreted as novel organisms, inflating diversity) generated as PCR artefacts due to non-specific primer binding will be removed. The quality filtered reads will then be grouped in Operational Taxonomic Units (OTUs) with 97% sequence similarity. Singleton (OTU with only 1 sequence) reads will be removed before taxonomic classification. Representative sequences from the OTUs will then be aligned to bacterial sequences from databases like Silva, Silva Gold and Green genes for classification of bacteria. Taxonomic abundances and Shannon diversity index (estimation of intra-individual diversity and evenness), β diversity and richness (Chao1 Index and Jaccard Index) will be performed using QIIME scripts (28).
Gut microbiome analysis will be done in National Institute of Biomedical Genomics, Kalyani, India.

Infant blood

We will collect 3 ml blood from infants at 3 and 6 months of age. The samples will be transported from the field to the "Clinical and Research laboratory" in SAS at 4°C on ice pack and centrifuged to extract serum and serum will be stored at -80°C for IGF-1, IGBP3 and CRP and AGP analysis. IGF-1 will be analysed by chemiluminescence and IGBP3 will be analysed by ELISA. CRP, AGP will be analysed by immunoturbidometry. These analyses will be done in St. John's Research Institute, Bangalore, India.

Future plan

It may be possible that the postulated pathways or the listed biomarkers may not be able to entirely explain the success or failure of the intervention package on growth failure. We will take consent from mothers to store her breast milk and baby's blood and stool to be used for future research, to understand growth failure. The stored samples will be deidentified.

Statistical Analysis

Intervention effect

The primary analysis will be based on intention to treat. Sociodemographic characteristics between groups A and D will be summarized (means, proportions). Any unbalanced sociodemographic characteristics across the groups that may affect the intervention impact on outcomes will be adjusted in multivariable models. We will examine the impact of the preconception, pregnancy, and early childhood care on biological outcomes at 6 months of age, compared to routine care. We will use generalized linear models (GLMs) of the Gaussian family with an identity-link function to calculate the difference in means and 95% confidence interval (CI) for the continuous outcomes. We will use GLM of the binomial or Poisson family with a log-link function to calculate the relative risk (RR) and 95% CI for binary outcomes.

Multivariable mixed-effect regression models will be done to show impact of the package of interventions on biological outcomes at 3 and 6 months of age, compared to group getting routine care using an unstructured covariance matrix (29).

All biomarkers will be assessed for normal distribution calculating skewness and kurtosis indices and plotting histogram, QQ plot. If any of the biomarkers show non-normal distribution, proper transformation will be done based on 'ladder' function in Stata (30).

We will truncate the outliers below 2.5th percentile and above 97.5th percentile for the Shannon index, species richness analysis (31). β diversity will be assessed by permutational multivariate analysis of variance (PERMANOVA), and analysis of similarities (ANOSIM) between the groups (32, 33). We will do Indicator species analyses (ISA) to identify taxa that will be different in intervention (Group A) and control (Group D) groups (34).

Mediation analysis

A counterfactual approach to mediation analysis will be done to determine the natural indirect effect (NIE) through each mediator, natural direct effect (NDE) through any other pathways outside of the mediator, and total effect of intervention on growth failure between 3-6 months of age (35). For each mother-infant dyad, we will assume that a mediator, for example systemic inflammation markers (CRP), can naturally take on different values depending on whether the mother-infant dyad received intervention or routine care. The NIE is the amount of the total effect of intervention on growth failure that can be explained by the mediator. The NDE

compares how growth failure will change comparing receiving interventions to receiving routine care; if we artificially fix systemic inflammation markers (CRP) for each infant to the natural value that we will expect the mother-infant dyad have received the routine care. Thus, the NDE explains the amount of the effect of intervention on growth failure that is not explained by the mediator. We will then calculate the proportion of the effect mediated by systemic inflammation (CRP) as the fraction of the total effect explained by the indirect effect. Separate mediation models will be developed for each of the mediators.

The natural direct and indirect effects will be estimated by fitting a generalized linear model with Poisson or binomial family and log link for the binary outcome, and a generalized linear model with Poisson or binomial family and log link for binary and generalized linear model with Gaussian family and identity link model for the continuous mediator, respectively.

The intervention (exposure) will be analyzed as a binary variable to reflect the comparison of Group A and Group D (Control). Growth failure will be defined using WHO growth velocity standards (36). Inadequate and adequate weight gain will be defined as 3 monthly (from 3 to 6 months of age) weight velocity <15th centile and ≥15th centile, respectively. Inadequate and adequate length gain will be defined as 3 monthly (from 3 to 6 months of age) length velocity <25th centile and ≥25th centile, respectively. The biomarkers will be analysed as continuous variables. The infant gestation and size will be analysed as continuous and as binary (term vs. preterm and AGA vs. SGA).

In addition to categorizing the growth failure of an infant into adequate and inadequate, we will also use superimposition by translation and rotation (SITAR) method to estimate the mean curve along with three subject specific random effects, namely, size, velocity and timing (37). We will use the 3 random effects i.e. size, velocity, and timing of peak separately to assess the mediating effects of the biological markers and infant gestation and size at birth of the intervention.

Table 3 Maternal Variables Measured in the Study

Maternal Variables	Enrolment	26-28 weeks of Gestation	35-37 weeks of Gestation	2 months postpartum	3 months postpartum	6 months postpartum
Age	✓					
Height	✓					
Body Mass Index (BMI)	✓					
Breast milk macro and micronutrients (% fat, % protein, Vitamin A, vitamin B12, vitamin D)					✓	✓
Human Oligosaccharides [2′-fucosyllactose (2′FL), 3-fucosyllactose (3FL), lacto-N-tetraose (LNT), lacto-N-fucopentaose I (LNFP I) and 3′-Sialyllactose (3′SL), 6′-Sialyllactose (6′SL)]					✓	✓
Micronutrients status (Vitamin A, D, B12, Folic Acid, Zinc, Selenium, Ferritin)			✓			
Serum Inflammatory Biomarkers (C-reactive protein, Alpha glycoprotein)			✓			
Weight gain during pregnancy	✓	✓	✓			
Estimated foetal weight by Ultrasonography		✓	✓			
Depressive symptoms by PHQ-9				✓		

Variables in blue will be captured in the current study

Table 4 Infant Variables Measured in the Study

Infant Variables	Birth	1m	3m	6 m
Weight	✓	✓	✓	✓
Length	✓	✓	✓	✓
Early Initiation of Breastfeeding	✓			
Exclusive Breastfeeding		✓	✓	✓(150-179 days)
Morbidity (Diarrhoea, Pneumonia, and febrile episode)		✓	✓	✓
Gut Microbiome		✓	✓	✓
Systemic inflammatory markers (CRP, AGP)		✓	✓	✓
Insulin like Growth factor-1, and Insulin-like Growth factor binding protein-3		✓	✓	✓

Variables in blue will be captured in the current study

A flow chart showing key stages of your research plans and indicates the location and timelines. This flowchart should be placed just before the references

Study Activities [Timelines]

Submission of Proposal to the Host Institute's Ethical Review Committee, Recruitment of Supporting Staff [June-July 2020]

↓

Ethics Committee Approval, Development of Study Tools, Training of Staff [September-October 2020]

↓

Selection of Mothers and Infants [November 2020 to September 2021]

↓

Collection of Breast Milk, Infant Stools and Blood Samples [November 2020 to September 2021]

↓

End of 6 Months Follow Up of Mothers and Infants [by September 2021]

↓

Preparatory activities –Protocol / SOP/ plan of analysis, Administrative issues – agreements with labs, data platforms
Sample transport and processing
[September 2021 to February 2022]

↓

Analyses of Breast Milk macro and micronutrients, Breast milk oligosaccharides, [March 2022 to February 2023]

↓

Infant gut microbiome analysis [March 2023 to February 2024]

↓

Infant systemic inflammation markers and IGF-1 [March 2024 to February 2025]

↓

Analysis and Report Writing [September 2025 to August 2026]

Training

Ethics in Biomedical research: Society for Applied Studies (Host Institute)

Mediation analysis methods and detailed plan of analysis: Dr. Partha Majumder, Dr. RM Pandey (at least once a week in the initial 6 months and then at least once a month)

Clinical and biological insights of the analysis plan: Dr. Rajiv Bahl, Dr. Nita Bhandari, Dr. Tor A. Strand, Dr. Sunita Taneja (monthly)

Analysis of Breast Milk macro and micronutrients and oligosaccharides at St. John's Research Institute (Dr. Anura Kurpad and Sarita): 2-3 week every 6 months [March 2022 to February 2023]

Gut microbiome analysis at National Institute of Biomedical Genomics (Dr. Souvik Mukherjee): 2-3 week every 6 months [March 2023 to February 2024]

Analysis of Infant systemic inflammation markers and IGF-1 at St. John's Research Institute (Dr. Anura Kurpad and Sarita): 2-3 week every 6 months [March 2024 to February 2025]

Activities highlighted in blue boxes will be started before September 2021, as all the infants will complete 6 months by that time and all these activities will be supported by the host institute.

References

1. Victora CG, de Onis M, Hallal PC, Blössner M, Shrimpton R. Worldwide timing of growth faltering: revisiting implications for interventions. Pediatrics. 2010;125(3):e473-80.
2. Patwari AK, Kumar S, Beard J. Undernutrition among infants less than 6 months of age: an underestimated public health problem in India. Maternal & child nutrition. 2015;11(1):119-26.
3. Young MF, Martorell R. The public health challenge of early growth failure in India. European journal of clinical nutrition. 2013;67(5):496-500.
4. Prendergast AJ, Humphrey JH. The stunting syndrome in developing countries. Paediatrics and international child health. 2014;34(4):250-65.
5. Olusanya BO, Renner JK. Predictors of growth velocity in early infancy in a resource-poor setting. Early human development. 2011;87(10):647-52.
6. Choudhary TS, Srivastava A, Chowdhury R, Taneja S, Bahl R, Martines J, et al. Severe wasting among Indian infants <6 months: Findings from the National Family Health Survey 4. Maternal & child nutrition. 2019;15(4):e12866.
7. Taneja S, Chowdhury R, Dhabhai N, Mazumder S, Upadhyay RP, Sharma S, et al. Impact of an integrated nutrition, health, water sanitation and hygiene, psychosocial care and support intervention package delivered during the pre- and peri-conception period and/or during pregnancy and early childhood on linear growth of infants in the first two years of life, birth outcomes and nutritional status of mothers: study protocol of a factorial, individually randomized controlled trial in India. Trials. 2020;21(1):127.
8. Dewey KG, Heinig MJ, Nommsen LA, Lonnerdal B. Maternal versus infant factors related to breast milk intake and residual milk volume: the DARLING study. Pediatrics. 1991;87(6):829-37.
9. Nommsen LA, Lovelady CA, Heinig MJ, Lönnerdal B, Dewey KG. Determinants of energy, protein, lipid, and lactose concentrations in human milk during the first 12 mo of lactation: the DARLING Study. The American journal of clinical nutrition. 1991;53(2):457-65.
10. Dror DK, Allen LH. Overview of Nutrients in Human Milk. Advances in nutrition (Bethesda, Md). 2018;9(suppl_1):278s-94s.
11. Prendergast AJ, Rukobo S, Chasekwa B, Mutasa K, Ntozini R, Mbuya MN, et al. Stunting is characterized by chronic inflammation in Zimbabwean infants. PloS one. 2014;9(2):e86928.
12. Pannaraj PS, Li F, Cerini C, Bender JM, Yang S, Rollie A, et al. Association Between Breast Milk Bacterial Communities and Establishment and Development of the Infant Gut Microbiome. JAMA pediatrics. 2017;171(7):647-54.
13. Robertson RC, Manges AR, Finlay BB, Prendergast AJ. The Human Microbiome and Child Growth - First 1000 Days and Beyond. Trends in microbiology. 2019;27(2):131-47.
14. Triantis V, Bode L, van Neerven RJJ. Immunological Effects of Human Milk Oligosaccharides. Front Pediatr. 2018;6:190-.
15. Charbonneau MR, O'Donnell D, Blanton LV, Totten SM, Davis JCC, Barratt MJ, et al. Sialylated Milk Oligosaccharides Promote Microbiota-Dependent Growth in Models of Infant Undernutrition. Cell. 2016;164(5):859-71.
16. Subramanian S, Huq S, Yatsunenko T, Haque R, Mahfuz M, Alam MA, et al. Persistent gut microbiota immaturity in malnourished Bangladeshi children. Nature. 2014;510(7505):417-21.
17. Blanton LV, Charbonneau MR, Salih T, Barratt MJ, Venkatesh S, Ilkaveya O, et al. Gut bacteria that prevent growth impairments transmitted by microbiota from malnourished children. Science (New York, NY). 2016;351(6275).
18. Yan J, Charles JF. Gut Microbiota and IGF-1. Calcified tissue international. 2018;102(4):406-14.
19. Kamng'ona AW, Young R, Arnold CD, Patson N, Jorgensen JM, Kortekangas E, et al. Provision of Lipid-Based Nutrient Supplements to Mothers During Pregnancy and 6 Months Postpartum and to Their Infants from 6 to 18 Months Promotes Infant Gut Microbiota Diversity at

18 Months of Age but Not Microbiota Maturation in a Rural Malawian Setting: Secondary Outcomes of a Randomized Trial. The Journal of Nutrition. 2020;150(4):918-28.
20. Schoemann AM, Boulton AJ, Short SD. Determining Power and Sample Size for Simple and Complex Mediation Models. 2017;8(4):379-86.
21. 354 S. [Available from: https://www.seca.com/en_my/products/all-products/product-details/seca354.html.
22. 417 S. [Available from: https://www.seca.com/en_my/products/all-products/product-details/seca417.html.
23. Miller EM, Aiello MO, Fujita M, Hinde K, Milligan L, Quinn EA. Field and laboratory methods in human milk research. American journal of human biology : the official journal of the Human Biology Council. 2013;25(1):1-11.
24. Hampel D, York ER, Allen LH. Ultra-performance liquid chromatography tandem mass-spectrometry (UPLC-MS/MS) for the rapid, simultaneous analysis of thiamin, riboflavin, flavin adenine dinucleotide, nicotinamide and pyridoxal in human milk. Journal of chromatography B, Analytical technologies in the biomedical and life sciences. 2012;903:7-13.
25. Wojcik KY, Rechtman DJ, Lee ML, Montoya A, Medo ET. Macronutrient analysis of a nationwide sample of donor breast milk. Journal of the American Dietetic Association. 2009;109(1):137-40.
26. Xu G, Davis JC, Goonatilleke E, Smilowitz JT, German JB, Lebrilla CB. Absolute Quantitation of Human Milk Oligosaccharides Reveals Phenotypic Variations during Lactation. J Nutr. 2017;147(1):117-24.
27. Williams GM, Leary SD, Ajami NJ, Chipper Keating S, Petrosin JF, Hamilton-Shield JP, et al. Gut microbiome analysis by post: Evaluation of the optimal method to collect stool samples from infants within a national cohort study. PloS one. 2019;14(6):e0216557-e.
28. Caporaso JG, Kuczynski J, Stombaugh J, Bittinger K, Bushman FD, Costello EK, et al. QIIME allows analysis of high-throughput community sequencing data. Nature methods. 2010;7(5):335-6.
29. Johnson W, Balakrishna N, Griffiths PL. Modeling physical growth using mixed effects models. American journal of physical anthropology. 2013;150(1):58-67.
30. Stata. ladder — Ladder of powers.
31. Dixon WJ. Simplified Estimation from Censored Normal Samples. The Annals of Mathematical Statistics. 1960;31(2):385-91.
32. Anderson MJ WD. PERMANOVA, ANOSIM, and the Mantel test in the face of heterogeneous dispersions: what null hypothesis are you testing? . Ecological Monographs. 2013;83:557–74.
33. MJ. A. Permutational Multivariate Analysis of Variance (PERMANOVA). Massey University, Auckland, New Zealand: John Wiley & Sons, Ltd; 2014-2017.
34. De Cáceres M LP, Moretti M. Improving indicator species analysis by combining groups of sites. Oikos. 2010;119:1674–84.
35. Richiardi L, Bellocco R, Zugna D. Mediation analysis in epidemiology: methods, interpretation and bias. International journal of epidemiology. 2013;42(5):1511-9.
36. The WHO Growth Standards [Internet]. 2006. Available from: https://www.who.int/childgrowth/en/.
37. Cole TJ, Donaldson MD, Ben-Shlomo Y. SITAR--a useful instrument for growth curve analysis. International journal of epidemiology. 2010;39(6):1558-66.
