# Supplementary material for: Impact of an integrated intervention package during preconception, pregnancy, and early childhood on inflammation, IGF-1, IGFBP3 during first 6 months of life: Findings from the WINGS randomized controlled trial
Source: PLOS Glob Public Health. 2025 Dec 15;5(12):e0005118. doi: 10.1371/journal.pgph.0005118 (PMC12704893; doi:10.1371/journal.pgph.0005118)
Supplement: Attachment — Submitted filename: Reviewer_Report_PLoS_Article.docx [file pgph.0005118.s003.docx]

**Title:**

Impact of an Integrated Intervention Package During Preconception, Pregnancy, and Early Childhood on Inflammation, IGF-1, and IGFBP3 During the First 6 Months of Life: Findings from the WINGS Randomized Controlled Trial

**1. Overall Assessment**

This study reports a well-designed randomized controlled trial. It investigates the impact of an integrated intervention on infant biomarkers related to inflammation and growth like CRP, AGP, IGF-1, IGFBP3. The research addresses a significant question in maternal and child health.

However, the discussion sections can be improved with detailed explanation on biological plausibility. Also, the implications of this study can be broadly elaborated.

**2. Originality and Relevance**

The research topic appears to be original and highly relevant. The novelty in this study is integrated interventions across different stages right from preconception to 2 years of early child development. The intervention is policy-relevant and aligns well as per Goal-2 and Goal-4 of SDG-2030. The concept is innovative and similar integrated frameworks are reported in the literature.

The specific distinct approach of this study needs to be articulated.

**3. Scientific Rigor and Methodology**

This randomized controlled design follows standard protocols and manuscript is well-aligned as per CONSORT guidelines.

Please elaborate on randomization process, blinding, and control of confounders. The sample size calculations appear to be powered for anthropometric assessments. For biomarker outcomes, sample size calculations need to be refined/justified.

**4. Results and Interpretation**

The results of this study report no significant differences in biomarkers between intervention and control groups.

The null findings can be discussed with possible biological explanations like timing of assessment, nutritional variability, breastfeeding. Subgroup analysis by maternal or infant characteristics can be helpful.

**5. Discussion and Implications**

There is a scope to elaborate the discussion section by linking the pathways of maternal interventions with infant biomarker responses. Implications of this study for public health, including integration into maternal and child health programs, can be discussed highlighting the need for long-term follow-up.

**6. Presentation and Clarity**

The manuscript is well-written and well-organized as per required guidelines. However, most of the references are quite older and references from 2022 onwards are missing.

More recent Citations can be included from year 2023-2025.

**7. Ethical and Data Considerations**

All the ethical procedures are described clearly including IEC and CTRI. Data availability through Open Access links is provided.

**8. Conclusion and Recommendation**

This well-executed trial can be good evidence for understanding the biological outcomes of integrated maternal-child interventions.

**Recommendation: Minor Revision.**
