# Supplementary material for: Impact of an integrated intervention package during preconception, pregnancy, and early childhood on inflammation, IGF-1, IGFBP3 during first 6 months of life: Findings from the WINGS randomized controlled trial
Source: PLOS Glob Public Health. 2025 Dec 15;5(12):e0005118. doi: 10.1371/journal.pgph.0005118 (PMC12704893; doi:10.1371/journal.pgph.0005118)
Supplement: Attachment — Submitted filename: Response to Reviewers_24.11.25.docx [file pgph.0005118.s005.docx]

**Response to Reviewers**

**Reviewer #1**: **This sub study was nested in a factorial randomized controlled trial (RCT) in women aged 18–30 years. Participants included in this study were randomized to receive either a preconception intervention package or routine care until early childhood. The design strategy involved a reasonable sample size justification to show superiority. The sample needed for the study objectives was well justified with power considerations. However, the investigators do note that the sample size, while adequate for detecting moderate effect sizes, may have been insufficient to identify smaller but clinically meaningful differences. The descriptives are informative as seen in Tables 1 and 2.**

*Response***:** Thanks for the overview.

**Comment 1**. **Please define IQR in the footnote of Table 2 or put a descriptive section in the ‘Analysis Plan’ paragraph**.

*Response*: We have added IQR definition in footnote of Table 2 and in the descriptive section in the analysis plan (Lines 240, 270-272).

**Generalized linear models (GLMs) with a Gaussian family and identity link function were used to estimate mean differences in CRP, AGP, IGF-1, and IGFBP3 concentrations. To estimate risk ratios for inflammatory status between infants in the intervention and routine care groups, GLMs with a binomial family and log link function were employed. Final models were adjusted for place of birth. There are several considerations needing clarification.

There are four endpoints. Therefore,

Comment 2. Some consideration of multiple comparison p-value adjustment should have been discussed.**

*Response:* Thanks for the suggestion. The objective of the current manuscript was to evaluate the effect of integrated interventions delivered from preconception, throughout pregnancy and early childhood on infant inflammation and IGF-1 and IGF-BP3 concentrations during first 6 months of life compared to routine care.

We *a priori* decided not to make alpha error adjustments for testing this set of related outcomes (related infant inflammatory status) pertinent to the hypothesized mechanisms of this multi-modal intervention. Such adjustments presume a universal null hypothesis that holds for all outcomes simultaneously but, because we cannot pre-specify which outcome or outcomes may most influence subsequent related policy-decisions, the universal null is of primary interest (*Rothman KJ. Epidemiology. 1990 Jan;1(1):43-6)*. Further, we envisaged that we would interpret that the intervention is efficacious only if it would have an effect on several primary outcomes. For instance, we would consider the intervention to be efficacious in improving inflammatory status if it showed an effect on any of the inflammatory markers.

We did not do multiple comparison because the outcomes were related to inflammatory markers.

**Also, with respect to model content,**

**Comment 3. Exactly how was adjustment by birthplace incorporated into the models?**

*Response:* Birthplace was included as one of the covariates in the GLM model as the proportion of birthplaces were different between intervention and control groups (Table 1). (Lines 258-260)

**The overall conclusions follow from the analyses performed and results seen in Table 3. The strengths and limitations are reasonably described in the ‘Discussion’ section. As an added point, however,

Comment 4. There is a gap between the manuscript text and the supplement supporting information proposal Version 2.0. Was there any attempt to explore the mediation analysis discussed in that proposal?**

*Response:* Thanks for the comment. In this manuscript, no attempts were made to quantify the mediating effect of the inflammatory markers or IGF-1 measured at 3 months of age on growth failure at 3 to 6 months. We will prepare a separate mediation analysis when all breast milk macro, micronutrient and HMO data are available.

**Reviewer #2**:

**Comment 1. Overall Assessment
This study reports a well-designed randomized controlled trial. It investigates the impact of an integrated intervention on infant biomarkers related to inflammation and growth like CRP, AGP, IGF-1, IGFBP3. The research addresses a significant question in maternal and child health.
However, the discussion sections can be improved with detailed explanation on biological plausibility. Also, the implications of this study can be broadly elaborated.**

*Response:* Thanks for the suggestion. We have now improved the explanation on biological plausibility, (Lines 289-294, 302-316) In the discussion we have mentioned

“Evidence from previous studies indicates that healthy children exhibit lower concentrations of CRP and AGP than malnourished children, and higher AGP levels have been associated with reduced height-for-age Z-scores.(11,12) Additionally, maternal micronutrient supplementation has been shown to alter maternal inflammatory biomarkers, which may subsequently affect metabolic outcomes in the offspring. (11,12) Building on this evidence our study found that CRP, AGP, and IGF-1 levels were similar between the intervention and routine care groups at both 3 and 6 months. IGFBP3 levels at 3 months were lower in the intervention group compared to the routine care group, but this difference was not sustained at 6 months.” (Lines 289-294)

“First, the prevalence of raised inflammatory markers in this population was relatively low, which may have limited the potential for further reduction in CRP and AGP even with an intensive intervention. Second, the IGF-1/IGFBP-3 axis in early infancy is strongly driven by age-related maturation, genetic programming and basic energy/protein sufficiency. Modest short-term improvements in nutrition, WASH, infection control, and psychosocial stimulation are unlikely to override these intrinsic trajectories within 3-6 months, attenuating the impact of the comprehensive intervention package.

The transient reduction in IGFBP3 at 3 months in the intervention group without a corresponding rise in IGF-1 likely reflects normal developmental variation or a small, short-lived change insufficient to alter total IGF-1 or tissue-level IGF-1 activity.” (Lines 302-316)

**Comment 2.** **Originality and Relevance
The research topic appears to be original and highly relevant. The novelty in this study is integrated interventions across different stages right from preconception to 2 years of early child development. The intervention is policy-relevant and aligns well as per Goal-2 and Goal-4 of SDG-2030. The concept is innovative and similar integrated frameworks are reported in the literature.
The specific distinct approach of this study needs to be articulated.**

*Response:* Thanks for the suggestion. We have now revised the originality and relevance of the study. (Lines 105-123).

“Building on this evidence, we designed the present study to evaluate the effects of the WINGS intervention on inflammatory markers and IGF-1/IGFBP3 concentrations during the first six months of life. By concurrently targeting maternal and infant health, nutrition, and environmental exposures during critical developmental windows, the intervention was expected to promote a healthier gut microbial milieu, attenuate systemic inflammation, and support more favourable growth factor profiles in infancy. (9)

We hypothesized that this integrated package would result in reduced levels of inflammatory markers and improved levels of IGF-1 and IGFBP3 in the intervention group compared to the routine care group. In doing so, this study aims not only to assess the clinical impact of WINGS but also to elucidate how multi-domain early-life interventions in low-resource settings modulate inflammation and growth factor pathways, thereby clarifying the biological mechanisms that link such interventions to improved growth and developmental outcomes. We chose 3 and 6 months as measurement time points to capture early postnatal windows as infant immune and endocrine systems are rapidly maturing and assessment at these intermediate endpoints help elucidate early biological pathways potentially underlying the later anthropometric effects observed at 24 months.” (Lines 105-123)

**Comment 3. Scientific Rigor and Methodology
This randomized controlled design follows standard protocols and manuscript is well-aligned as per CONSORT guidelines.
Please elaborate on randomization process, blinding, and control of confounders. The sample size calculations appear to be powered for anthropometric assessments. For biomarker outcomes, sample size calculations need to be refined/justified.**

*Response:* Thanks. We have elaborated on the randomization process. (Lines 143,144, 155-158).

“Women were randomised using permuted blocks and stratified by maternal height (<150 cm and>= 150 cm). Group allocation (1:1) was through a web based system.” (Lines 143,144)

“All women were counseled on adequate diets, positive thinking, problem-solving skills, and menstrual and hand hygiene. We could not mask participants and teams because of the nature of the interventions. Outcomes were assessed by an independent team not involved in delivering interventions or aware of the group allocation before measurements.” (Lines 155-158).

The Sample size has been calculated based on biomarker outcomes. As mentioned in the manuscript:

“Sample Size Calculation: Few intervention studies have systematically examined the infant blood markers of inflammation and growth during the first six months of life. In the WINGS study, we hypothesized a 0.2 standard deviation (SD) increase in Length-for-Age Z-score (LAZ) and a 25% relative reduction in the incidence of stunting at 24 months in the group receiving interventions during preconception, pregnancy, and early childhood (Group A), compared to the group receiving routine care (Group D) (5). For the present study, we hypothesized that the intervention package would result in increased IGF-1 levels and reduced inflammatory markers, such as CRP, with an anticipated effect size equal to or greater than that observed in the WINGS study. Assuming a corresponding 0.3 SD decrease of CRP in the intervention group with the power at 80% and 95% confidence level, we calculated that a total of 352 infant blood samples would be required – 176 in the intervention group and 176 in the routine care group.” (Line 203-214)

**Comment 4.** **Results and Interpretation
The results of this study report no significant differences in biomarkers between intervention and control groups.
The null findings can be discussed with possible biological explanations like timing of assessment, nutritional variability, breastfeeding. Subgroup analysis by maternal or infant characteristics can be helpful.**

*Response:* Thanks for the suggestion. We now updated the discussion based on the suggestions. (Lines 289-294, 302-316, 332-344)

“Evidence from previous studies indicates that healthy children exhibit lower concentrations of CRP and AGP than malnourished children, and higher AGP levels have been associated with reduced height-for-age Z-scores.(11,12) Additionally, maternal micronutrient supplementation has been shown to alter maternal inflammatory biomarkers, which may subsequently affect metabolic outcomes in the offspring. (11,12) Building on this evidence our study found that CRP, AGP, and IGF-1 levels were similar between the intervention and routine care groups at both 3 and 6 months. IGFBP3 levels at 3 months were lower in the intervention group compared to the routine care group, but this difference was not sustained at 6 months.” (Lines 289-294)

“First, the prevalence of raised inflammatory markers in this population was relatively low, which may have limited the potential for further reduction in CRP and AGP even with an intensive intervention. Second, the IGF-1/IGFBP-3 axis in early infancy is strongly driven by age-related maturation, genetic programming and basic energy/protein sufficiency. Modest short-term improvements in nutrition, WASH, infection control, and psychosocial stimulation are unlikely to override these intrinsic trajectories within 3-6 months, attenuating the impact of the comprehensive intervention package.

The transient reduction in IGFBP3 at 3 months in the intervention group without a corresponding rise in IGF-1 likely reflects normal developmental variation or a small, short-lived change insufficient to alter total IGF-1 or tissue-level IGF-1 activity.” (Lines 302-316)

“Moreover, early-life inflammation is shaped by a complex interplay of perinatal factors, infections, gut health, microbiota, and oxidative stress, so systemic markers such as CRP and AGP may miss subtle or localized benefits of the intervention. The timing and duration of the intervention, while spanning critical developmental windows, may not have been sufficient to elicit measurable changes in systemic biomarkers within the first six months of life. Any effects of improved nutrition and environment on growth and endocrine function may emerge only later in infancy or childhood, and our intervention may have enhanced overall adequacy rather than the specific nutrient profiles (e.g., particular protein sources or micronutrients) known to more strongly modulate IGF-1 and IGFBP-3. Conclusions based solely on data from 3 and 6 months may be inappropriate. It is also possible that the benefits of the intervention on inflammation and growth factors may emerge later in childhood, as has been observed in some longitudinal studies. (16) However, that again will be subject to varying degrees of confounding factors over a long time period that can alter the levels of the inflammatory markers. (17)” (Lines 332-344)

We did not have a prior sub-group analysis plan.

**Comment 5.** **Discussion and Implications
There is a scope to elaborate the discussion section by linking the pathways of maternal interventions with infant biomarker responses. Implications of this study for public health, including integration into maternal and child health programs, can be discussed highlighting need for long-term follow-up.**

*Response:* Thanks for your suggestion. We have elaborated on the pathways affecting the inflammatory responses and highlighted the implications of the study and the need for long-term follow-up to explore the changes in inflammatory processes. We have also added the relevance of the study in the Conclusion section. (Lines 332-344, 359-361).

“Moreover, early-life inflammation is shaped by a complex interplay of perinatal factors, infections, gut health, microbiota, and oxidative stress, so systemic markers such as CRP and AGP may miss subtle or localized benefits of the intervention. The timing and duration of the intervention, while spanning critical developmental windows, may not have been sufficient to elicit measurable changes in systemic biomarkers within the first six months of life. Any effects of improved nutrition and environment on growth and endocrine function may emerge only later in infancy or childhood, and our intervention may have enhanced overall adequacy rather than the specific nutrient profiles (e.g., particular protein sources or micronutrients) known to more strongly modulate IGF-1 and IGFBP-3. Conclusions based solely on data from 3 and 6 months may be inappropriate. It is also possible that the benefits of the intervention on inflammation and growth factors may emerge later in childhood, as has been observed in some longitudinal studies. (16) However, that again will be subject to varying degrees of confounding factors over a long time period that can alter the levels of the inflammatory markers. (17)” (Lines 332-344)

“Additionally, the study was conducted in a single urban setting in India, which may limit generalizability to other populations. Replicating the study in diverse socioeconomic and cultural settings would strengthen the evidence base and enable more conclusive interpretations. (183)

Conclusion

While early evidence points to the therapeutic effect of early nutritional and health interventions, this is one of the few prospective studies that have tried to evaluate the effect of the interventions on inflammatory processes.” (Lines 359-361).

**Comment 6. Presentation and Clarity
The manuscript is well-written and well-organized as per required guidelines. However, most of the references are quite older and references from 2022 onwards are missing.
More recent Citations can be included from year 2023-2025.**

Response: Thanks for the relevant suggestion. We have added the references to the following studies conducted on and after 2023.

1. Wunderle C, Stumpf F, Schuetz P. Inflammation and response to nutrition interventions. Journal of Parenteral and Enteral Nutrition. 2024 Jan;48(1):27-36. (*Ref no. 17*)
2. DeBoer MD, Elwood SE, Platts-Mills JA, McDermid JM, Scharf RJ, McQuade ET, Jatosh S, Houpt ER, Mduma E. Association of Circulating Biomarkers with Growth and Cognitive Development in Rural Tanzania: A Secondary Analysis of the Early Life Interventions in Childhood Growth and Development In Tanzania (ELICIT) Study. The Journal of Nutrition. 2023 May 1;153(5):1453-60. (*Ref no. 16*)
3. DeBoer MD, Elwood SE, Platts-Mills JA, McDermid JM, Scharf RJ, McQuade ET, Jatosh S, Houpt ER, Mduma E. Association of Circulating Biomarkers with Growth and Cognitive Development in Rural Tanzania: A Secondary Analysis of the Early Life Interventions in Childhood Growth and Development In Tanzania (ELICIT) Study. The Journal of Nutrition. 2023 May 1;153(5):1453-60. (*Ref no. 7*)
4. Nigam M, Devi K, Coutinho HD, Mishra AP. Exploration of gut microbiome and inflammation: A review on key signalling pathways. Cellular Signalling. 2024 Mar 16:111140. (*Ref no. 9*)

**Comment 7. Ethical and Data Considerations
All the ethical procedures are described clearly including IEC and CTRI. Data availability through Open Access links is provided**.

*Response:* Thanks

**Comment 8. Conclusion and Recommendation
This well-executed trial can be good evidence for understanding the biological outcomes of integrated maternal-child interventions.**

*Response:* Thanks

**Reviewer #3: This study is a secondary analysis of the WINGS factorial randomized controlled trial evaluating the effects of a multidomain, integrated intervention delivered from preconception through early childhood on infant biomarkers of inflammation and growth (CRP, AGP, IGF-1, IGFBP3) at 3 and 6 months of age. This study links the integrated intervention to specific changes in inflammatory and growth-related biomarkers like CRP, AGP, IGF-1 and IGFBP3. The study addressed the biologically relevant and policy-important question related to early-life interventions in low-resource settings
The findings indicate no significant differences in these biomarkers between the intervention and control groups, except for a transient decrease in IGFBP3 at 3 months, which was not sustained at 6 months. The authors conclude that while the intervention improved growth outcomes in the parent trial, it did not significantly influence early-life inflammation or IGF axis biomarkers.
The manuscript is well-written, clearly articulated and follows the required CONSORT Guidelines.
Major Comments
Comment 1. Rationale and Framing
• Biological rationale connecting integrated maternal–child interventions (nutrition, WASH, psychosocial care) with the specific biomarkers studied (CRP, AGP, IGF-1, IGFBP3), needs clarity**

*Response:* Thanks for the relevant suggestions. We have revised the rationale in the introduction section. (Lines 105-123). The explanation on biological rationale and the need for connecting the interventions with biomarkers has been further elaborated in the discussion section. (Lines 289-293, 302-316, 332-344)

**• Clarify why these markers and 3- and 6-month time points were selected, especially since primary growth outcomes were reported at 24 months in the main WINGS paper.**

*Response:* Thanks for the comment. We chose 3 and 6 months as measurement time points to capture early postnatal windows when infant immune and endocrine systems are rapidly maturing, and when the WINGS intervention, initiated in preconception, continued throughout pregnancy and early childhood, would be expected to exert its most immediate biological effects on inflammation and growth-factor dynamics. In contrast, the primary growth outcomes at 24 months reported in the main WINGS paper were intended to reflect the cumulative, long-term impact of the intervention on linear growth and child development. Thus, the 3- and 6-month biomarker assessments were designed as mechanistic, intermediate endpoints to help elucidate early biological pathways potentially underlying the later anthropometric effects observed at 24 months.

We have added this clarification in the manuscript. (Lines 120-123)

“We chose 3 and 6 months as measurement time points to capture early postnatal windows as infant immune and endocrine systems are rapidly maturing and assessment at these intermediate endpoints help elucidate early biological pathways potentially underlying the later anthropometric effects observed at 24 months.”

**• A concise conceptual model or figure showing hypothesized pathways could help readers follow the mechanistic logic.**

Response: Thanks for the suggestions. We have now added a conceptual framework. Figure 2. Conceptual framework: Postulated pathways of intervention effect to reduce growth faltering first 6 months of age. (Lines 256,257)

Conceptual framework: Postulated pathways of intervention effect to reduce growth faltering first 6 months of age

**2. Study Power and Sample
• The power calculation is based on CRP only. Please justify the adequacy of the sample size for detecting meaningful differences in IGF-1 and IGFBP3, given their biological variability in infancy**.
**• Power calculations are based on LAZ outcomes from the primary WINGS study rather than biomarker data. This needs justification.**

*Response*: Thanks for the suggestion. We would like to clarify that the sample size calculations for this sub-study were conducted based on the biomarker outcomes. The relevant details have been incorporated in lines 204–214 of the revised manuscript.

“Sample Size Calculation: Few intervention studies have systematically examined the infant blood markers of inflammation and growth during the first six months of life. In the WINGS study, we hypothesized a 0.2 standard deviation (SD) increase in Length-for-Age Z-score (LAZ) and a 25% relative reduction in the incidence of stunting at 24 months in the group receiving interventions during preconception, pregnancy, and early childhood (Group A), compared to the group receiving routine care (Group D) (5). For the present study, we hypothesized that the intervention package would result in increased IGF-1 levels and reduced inflammatory markers, such as CRP, with an anticipated effect size equal to or greater than that observed in the WINGS study. Assuming a corresponding 0.3 SD decrease of CRP in the intervention group with the power at 80% and 95% confidence level, we calculated that a total of 352 infant blood samples would be required – 176 in the intervention group and 176 in the routine care group.” (Line 203-214)

**3. Statistical Analysis and results
• Tables 2 and 3 could be simplified to highlight group comparisons more effectively.**

*Response:* Thanks for the suggestions. We have now formatted the tables.

**• Adjustment only for the place of delivery seems limited.**

*Response:* Birthplace was included as one of the covariates in the GLM model as the proportion of birthplaces was different between intervention and control groups.

Table 1 depicts that the distribution of other variables were similar between the two groups.

**• The author may consider other covariates, such as mothers’ BMI, socioeconomic indicators, or exposure to infections, in the analysis. In case they are intentionally excluded from the analysis, explain their exclusion.**

*Response:* Thanks for the suggestion. The variables suggested, such as maternal BMI, socioeconomic indicators, and exposure to infections, were comparable between the intervention and control groups at baseline, as shown in Table 1. Since there were no significant differences between groups, these covariates were not included in the final model.

**• It would be useful to include effect size interpretation (e.g., percentage change or standardized mean difference) to better convey the biological relevance of null findings.**

*Response:* Thanks for the suggestion. We have now included the same in the results. (Lines 271-272, 282)

**4. Interpretation of Findings
• However, cautious interpretation of the null findings is needed. Aspects such as biological plausibility, contextual limitations, and future implications for longitudinal research require further elaboration.
• The discussion acknowledges the absence of significant effects, but can be deepened if the authors discuss the following issues
o Address low baseline inflammation as a potential ceiling effect.
o Note that intervention effects might appear later in life (after 6 months).
o Acknowledge that non-inflammatory mechanisms (caregiving, infection prevention, psychosocial stimulation) might explain the positive growth outcomes in the primary trial.**

*Response*: Thanks for the suggestion. We have now modified the discussion according to the suggestions. (Lines 289-293, 302-316, 332-344)

**• Expand the comparison with similar trials—such as SHINE (Zimbabwe), ELICIT (Tanzania), and MAL-ED studies—that examined inflammation and growth factor pathways**.

*Response*: Thanks for the suggestion. We have now included the comparative findings of the studies. (Lines 289-291, 322-323, 332-344)

“Evidence from previous studies indicates that healthy children exhibit lower concentrations of CRP and AGP than malnourished children, and higher AGP levels have been associated with reduced height-for-age Z-scores.(11,12) Additionally, maternal micronutrient supplementation has been shown to alter maternal inflammatory biomarkers, which may subsequently affect metabolic outcomes in the offspring. (11,12) Building on this evidence our study found that CRP, AGP, and IGF-1 levels were similar between the intervention and routine care groups at both 3 and 6 months. IGFBP3 levels at 3 months were lower in the intervention group compared to the routine care group, but this difference was not sustained at 6 months.” (Lines 289-294)

“IGFBP3 is the primary binding protein for IGF-1, playing a crucial role in regulating IGF-1 bioavailability and activity. While lower IGFBP3 could theoretically enhance IGF-1 action, we did not observe corresponding increases in IGF-1 concentrations at this early time point. This case control study in Zimbabwe found that IGF-1 and IGFBP-3 decreased with time from 3 months of age among both stunted and non-stunted children. (4)” (Lines 322-323)

“Moreover, early-life inflammation is shaped by a complex interplay of perinatal factors, infections, gut health, microbiota, and oxidative stress, so systemic markers such as CRP and AGP may miss subtle or localized benefits of the intervention. The timing and duration of the intervention, while spanning critical developmental windows, may not have been sufficient to elicit measurable changes in systemic biomarkers within the first six months of life. Any effects of improved nutrition and environment on growth and endocrine function may emerge only later in infancy or childhood, and our intervention may have enhanced overall adequacy rather than the specific nutrient profiles (e.g., particular protein sources or micronutrients) known to more strongly modulate IGF-1 and IGFBP-3. Conclusions based solely on data from 3 and 6 months may be inappropriate. It is also possible that the benefits of the intervention on inflammation and growth factors may emerge later in childhood, as has been observed in some longitudinal studies. (16) However, that again will be subject to varying degrees of confounding factors over a long time period that can alter the levels of the inflammatory markers. (17)” (Lines 332-344)

**• The trial was conducted in a single urban Indian setting, which limits extrapolation to rural or diverse socioeconomic contexts. The discussion should acknowledge this limitation more explicitly and suggest strategies for replication in varied environments.**

*Response*: Thanks for the suggestion. We have now included this as one of the limitations and suggested strategies for replication in varied environments, (Lines 354-356)

**5. Policy and Program Implications
• The conclusion is based on the non-significant findings of biomarkers. Whereas the short duration of biomarker assessment may oversimplify complex biological processes. More elaborate discussion is needed on possible confounders like infections, duration, and type of breastfeeding.**

*Response*: Thanks for the suggestion. We have made the suggested changes. (Lines 355-361)

**Minor Comments
1. Abstract: Conclude with a stronger statement about contribution: e.g., “These findings add to the understanding of biological mechanisms underlying integrated early-life interventions in LMICs.”**

*Response:* Thanks for the suggestion. We have revised accordingly. (Lines 367,368)

**2. Tables: Present only adjusted results in the main text; unadjusted data may be submitted as supplementary files. Ensure all tables include units (mg/L, ng/mL) and consistent decimal formatting.**

*Response:* Thanks for noting the units. We have defined CRP in mg/L, AGP in g/L, IGF-1 in ng/mL and IGFBP3 in ng/mL.

**3. CONSORT Diagram: Please include the number of exclusions, losses to follow-up, and reasons for non-participation in Figure 1 for transparency.**

*Response:* The exclusions, number of losses to follow-up at each stage and the reasons of non-participation has been already mentioned in the CONSORT diagram.

**4. Discussion: Add a short note acknowledging that biomarker variability in early infancy is high and may obscure subtle intervention effects**.

*Response*: Thanks. We have included one sentence in the discussion. (Lines 332,344)

**5. References: Consider citing more recent literature (published within the last 3 years) that links microbiome–inflammation–growth relationships in infants.**

*Response*: Thanks for the suggestion. We have included the most recent (3 years) references.

1. Wunderle C, Stumpf F, Schuetz P. Inflammation and response to nutrition interventions. Journal of Parenteral and Enteral Nutrition. 2024 Jan;48(1):27-36. (*Ref no. 17*)
2. DeBoer MD, Elwood SE, Platts-Mills JA, McDermid JM, Scharf RJ, McQuade ET, Jatosh S, Houpt ER, Mduma E. Association of Circulating Biomarkers with Growth and Cognitive Development in Rural Tanzania: A Secondary Analysis of the Early Life Interventions in Childhood Growth and Development In Tanzania (ELICIT) Study. The Journal of Nutrition. 2023 May 1;153(5):1453-60. (*Ref no. 16*)
3. DeBoer MD, Elwood SE, Platts-Mills JA, McDermid JM, Scharf RJ, McQuade ET, Jatosh S, Houpt ER, Mduma E. Association of Circulating Biomarkers with Growth and Cognitive Development in Rural Tanzania: A Secondary Analysis of the Early Life Interventions in Childhood Growth and Development In Tanzania (ELICIT) Study. The Journal of Nutrition. 2023 May 1;153(5):1453-60. (*Ref no. 7*)
4. Nigam M, Devi K, Coutinho HD, Mishra AP. Exploration of gut microbiome and inflammation: A review on key signalling pathways. Cellular Signalling. 2024 Mar 16:111140. (*Ref no. 9*)

**6. Language and Formatting: Ensure consistency in abbreviations (e.g., IGFBP3 vs IGF-BP3). Use consistent phrasing for “preconception, pregnancy, and early childhood interventions, growth-related biomarkers, and growth factor profiles” throughout.**

*Response:* Thanks for the suggestions. We have ensured consistency throughout the manuscript as IGFBP3.

**Overall Recommendations: Minor–to–Moderate Revision
This is a robust, well-implemented study addressing an important mechanistic question within global child health. Although the results are null, they offer valuable insights into early-life biology and integrated program evaluation. Strengthening the biological framing, contextual discussion, and presentation of adjusted analyses will substantially enhance the manuscript’s impact and readability.**

Response: Thanks.
